# Health Risk Assessment Based on Source Identification of Heavy Metal(loid)s: A Case Study of Surface Water in the Lijiang River, China

**DOI:** 10.3390/toxics10120726

**Published:** 2022-11-25

**Authors:** Yu Wang, Cunlin Xin, Shi Yu, Yincai Xie, Wanjun Zhang, Rongjie Fu

**Affiliations:** 1 College of Geography and Environmental Science, Northwest Normal University, Lanzhou 730070, China; 2 Key Laboratory of Resource Environment and Sustainable Development of Oasis, Lanzhou 730070, China; 3 Key Laboratory of Karst Dynamics, MNR & GZAR, Institute of Karst Geology, Chinese Academy of Geological Sciences, Guilin 541004, China; 4 International Research Center on Karst under the Auspices of UNESCO, Guilin 541004, China

**Keywords:** surface water, Lijiang River, heavy metal(loid)s, health risk assessment, source identification, ICP-MS

## Abstract

In this study, 24 surface water samples were collected from the main trunk/tributary of the Lijiang River during the wet season (April) and the dry season (December) in 2021. The total concentration of 11 heavy metal(loid)s (Al, Cu, Pb, Zn, Cr, Ni, Co, Cd, Mn, As, and Hg) was determined to investigate their physicochemical properties and spatial-temporal distribution characteristics. The heavy metal evaluation index (HEI) and the positive matrix factorization (PMF) model were employed to evaluate water quality and to reveal quantitatively identified pollution sources for further investigation to obtain a health risk assessment using the hazard index (HI) and carcinogenic risk (CR) of various pollution sources. The mean concentrations of heavy metal(loid)s in surface water in the wet and dry seasons were ranked as: Al > Mn > Zn > Ni > Cd > Cr > Cu > As >Hg = Pb > Co, with the mean concentration of Hg being higher than the national Class II surface water environmental quality standard (GB3838-2002). In terms of time scale, the concentration of most heavy metal(loid)s was higher in the wet season; most heavy metal(loid)s were distributed mainly in the midstream area. HEI index indicated that the main water quality status was “slightly affected” in the study area. Five potential sources of pollution were obtained from the PMF model, including industrial activities, traffic sources, agricultural activities, domestic waste emissions, and natural resources. The source-oriented risk assessment indicated that the largest contributions of HI and CR were agricultural sources in the Lijiang River. This study provides a “target” for the precise control of pollution sources, which has a broad impact on improving the fine management of the water environment in the basin.

## 1. Introduction

Rivers supply water for people’s productivity and livelihood, which is of great significance for the development of anthropogenic society and the maintenance of ecological balance [1,2]. However, heavy metal(loid) contamination in rivers has become a major threat to the health of regional ecosystems [3]. Generally, heavy metal(loid)s in rivers originate from natural processes (such as rock weathering and atmospheric deposition) and anthropogenic activities (such as fossil fuel combustion, transportation, industrial production, fertilizers, and pesticides) [4,5]. Physical and chemical properties of the water and its surrounding environment could be influenced by heavy metal(loid)s [3,6], which could also pose potential threats to human health via dermal absorption and direct ingestion [7]. For example, consumption of Cd-contaminated surface water for long periods of time could induce anemia, nephropathy, and cancers of the digestive system [8]; long-term exposure to As may cause visceral cancers and neurological and cardiovascular diseases [9]. In addition, migration and degradation of heavy metal(loid)s in aquatic environments could easily spread to other regions [10,11]. Therefore, the evaluation of pollution sources and health risks of heavy metal(loid)s in surface river water has become an active research topic for future investigation of the aquatic environment.

**Figure 1:**
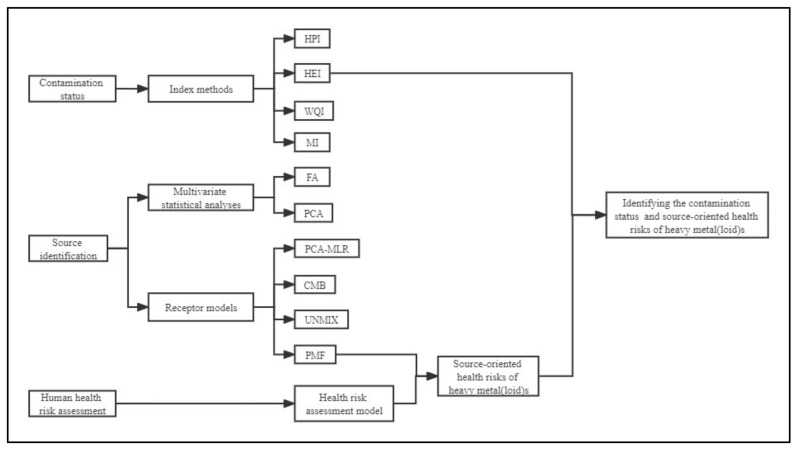
Research Methods and Ideas.

Identifying the contamination status, sources, and health risks of heavy metal(loid)s is a priority for reducing the risk of heavy metal(loid)s. Presently, index methods such as the heavy metal pollution index (HPI) [4,12,13], heavy metal evaluation index (HEI) [14,15], water quality index (WQI) [16,17], metal index (MI) [18,19], single-factor pollution index, and Nemerow’s pollution index [20], have been extensively adopted to appraise the status of heavy metal(loid)s contamination in surface water, respectively. Multivariate statistical analyses, for instance, correlation analysis, factor analysis (FA), and principal component analysis (PCA), have been employed to the identification of sources of heavy metal(loid)s contamination in surface water [20,21,22]. Receptor models, including principal component analysis–multiple linear regressions (PCA-MLR), chemical mass balance (CMB), UNMIX, and positive matrix factorization (PMF), have been applied to the quantitative source apportionment of heavy metal(loid)s in surface water [2,23,24]. Additionally, the carcinogenic and non-carcinogenic risks of heavy metal(loid)s in surface water have been appraised via health risk assessment models (Figure 1) [25]. Giri et al. studied the sources of groundwater metal contamination in the Subarnarekha River basin in India and found that they were related mainly to geological formations of the crust and basin, mining activities, traffic pollution, and geogenesis, with contributions of 34.04%, 22.34%, 14.67%, and 13.93%, respectively [26]. Ismail et al. studied source identification of heavy metal pollution in surface water along the Straits of Malacca and found that Cu and Cr dominate the source apportionment in northern region with a total variance of 57.62% and are identified with mining and shipping activities. The high level of Pb concentration in the southern region is attributed to land-based pollution originating from vehicular emission, with a total variance of 59.43% [27]. Nevertheless, the majority of previous studies have focused on the quantitative identification of potential sources of heavy metal(loid)s in water, with less attention to the health risks of specific sources. Sheng et al. found that the accumulation of heavy metal(loid)s in the aquatic environment could not pose a high risk to human health, while different sources may have a significant influence on the toxicity, distribution, and occurrence of heavy metal(loid)s [9]. Consequently, source-oriented health risks of heavy metal(loid)s in surface water need to be appraised to protect human health.

The Lijiang River is a surface river of karst characterized by carbonate strata [28]. Owing to its beautiful karst landscape and limpid water quality, the Lijiang River has become the most valuable tourism and water resource for tourists and local residents [29]. Nevertheless, with the rapid development of urbanization and tourism along the Lijiang River, a large amount of heavy metal(loid)s, organic compounds, and other contaminants are continuously drained into the river [30], causing effects of varying degrees on the water quality of the river [31]. Hence, it is necessary to determine the heavy metal(loid)s in surface water of the Lijiang River. The main methods for the determination of elements by different analytical techniques are atomic fluorescence spectrometry, atomic absorption spectrometry, X-ray fluorescence spectrometry, and inductively coupled plasma mass spectrometry (ICP-MS); due to the analytical possibilities and well-established analytical calibration procedures, ICP-MS is the most advanced and commonly used analytical method [32,33,34]. The assessment of the pollution status, sources, and health risks based on the measured concentrations of heavy metal(loid)s in the Li River basin is of great importance for aquatic environmental protection.

In the present research, the physicochemical properties, spatial-temporal distribution characteristics, and water quality conditions of 11 heavy metal(loid)s (Al, Cu, Pb, Zn, Cr, Ni, Co, Cd, Mn, As, and Hg) in the wet season (April) and dry season (December) of the main trunk/tributary of the Lijiang River Basin in 2021 were investigated, and a comprehensive method was proposed for source identification and source-oriented health risk assessment of heavy metal(loid)s using the PMF model and human health risk assessment model. Consequently, the primary goals of the current research were to (1) analyze the spatial-temporal distribution characteristics of heavy metal(loid)s in the surface water of the Lijiang River; (2) evaluate the water quality of the surface water; (3) analyze the possible sources of the heavy metal(loid)s in the surface water; (4) calculation of the non-carcinogenic and carcinogenic risks of heavy metal(loid)s to surrounding residents in the Lijiang River; and (5) import the results of source identification into the environmental risk model and estimate the source-oriented health risk.

## 2. Materials and Methods

### 2.1. Study Area

**Figure 2:**
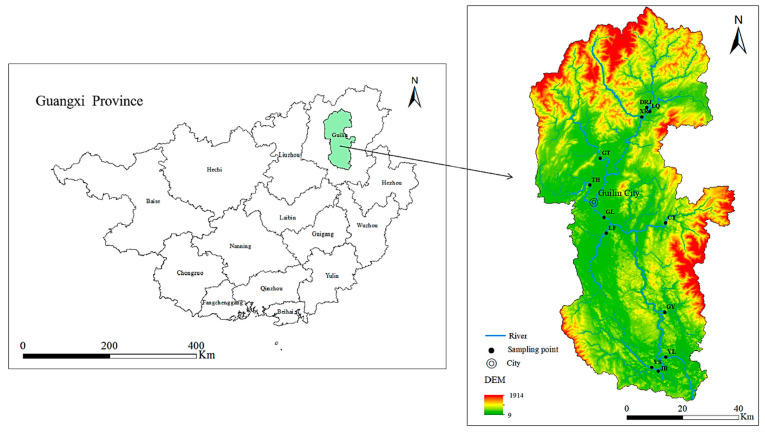
Study area and distribution of sampling points.

The Lijiang River is located in the northeast of Guangxi Province and belongs to the upper reaches of the Guijiang River of the Pearl River system (Figure 2). It originates from Mao’er Mountain in Xing’an County, Guangxi, flows through Xing’an County, Lingchuan County, Guilin City, and Yangshuo County from north to south, and joins the Guijiang River in Pingle County. The study area has a subtropical monsoon climate, the annual average temperature and rainfall are approximately 19.2 °C and 1890.4 mm, respectively [35].

The Lijiang River is a rain-sourced mountain river. The runoff is formed by rainfall, and the annual runoff is relatively abundant, but the annual and inter-annual distribution is extremely uneven. The wet season is from March to August, and the runoff accounts for nearly 80% of the annual runoff, of which May to June account for 40% of the annual runoff. The dry season is from September to February; the runoff in January, the driest month, accounts for only 2% of the annual runoff. The terrain of the study area is dominated by mountains and plains, with hills, basins, and karst landforms, and it is a low to medium mountainous and hilly terrain, with the Li River as the axis of the entire watershed, which is distributed in a narrow north–south direction [29]. In addition, the Li River provides an important resource for agriculture, industrial activities, and drinking water for local residents, and understanding the risks and sources of its contamination is important for water conservation.

### 2.2. Collection and Analyses of Water Samples

In this study, 24 surface water samples were collected in the main trunk/tributary of the Lijiang River Basin (Figure 2), including upstream points: LQ, DRJ, and XRJ; midstream points: GT, TH, GL, LF, and CT; and downstream points: GY, YL, JB, and YS (Table 1). According to the hydrological conditions of the basin, the sampling was divided into the wet season (April) and the dry season (December). All samples were collected in polyethylene bottles, and the sampling containers were rinsed three times with river water at the sampling site before sampling. After the water samples were collected, HNO_3_ was added to make the pH ≤ 2. At the same time, the water samples were tested for pH, water temperature (T), electrical conductivity (EC), and dissolved oxygen (DO) using a Manta2TM4.0 multi-parameter water quality meter from Eureka, Austin, TX, USA. The collected water samples were sent to the Testing Center of the Institute of Karst Geology, Chinese Academy of Geological Sciences, for analysis and testing. After taking them back to the laboratory, they were immediately filtered with a 0.45 μm filter and stored in a refrigerator at 4 °C away from light. The concentrations of the heavy metal(loid)s Al, Cu, Pb, Zn, Cr, Ni, Co, Cd, Mn, As, and Hg were determined using inductively coupled plasma mass spectrometry (ICP-MS) according to the Standard Examination Methods for Drinking Water-Metal Parameters (GB/T5750.6-2006). In this test, the standard substances provided by the National Standards Center were used for on-machine determination, so as to ensure the validity of the data and the accuracy of the analysis method. The standard deviations of the test results of all elements were within 10%. The chemical reagents used in the analysis process were all excellent grade pure, and the glassware used was soaked in the prepared 10% nitric acid solution for more than 24 h.

### 2.3. Assessment Method

#### 2.3.1. Evaluation of Surface Water Quality Based on Heavy Metal(loid)s

In order to appraise the comprehensive effect of heavy metal(loid)s on water quality, the heavy metal evaluation index (HEI) was employed to explain the pollution index and degree of contamination [36,37], which was calculated as follows:

(1) $$\mathrm{HEI}=\overset{i=n}{\underset{i=1}{\sum }}\frac{C_{i}}{MAC_{i}}$$

where *C_i_* is the concentration of *i*-th heavy metal(loid)s (ug·L^−1^), and *MAC_i_* is the highest standard allowable level, which is adopted from the National Environmental Quality Standards for Surface Water (GB3838-2002) [38]. If the metal concentration was below the undetectable level, the analytical detection limit was used rather than zero in this study.

This index is classified into six categories [39], which include HEI < 0.3 (very pure), 0.3 < HEI < 1.0 (poor), 1.0 < HEI < 2.0 (slightly affected), 2.0 < HEI < 4.0 (moderately affected), 4.0 < HEI < 6.0 (strong affected), and HEI > 6.0 (seriously affected).

#### 2.3.2. Human Health Risk Assessment

Human health risk values could evaluate and forecast the hazard to human health resulting from various environmental contaminants and the probability of their occurrence [40]. In this research, the health risk assessment model recommended by the US Environmental Protection Agency was employed as the assessment method for the health risk of humans exposed to heavy metal(loid)s in surface water of the Lijiang River [41]. It is measured by:

(2) $$ADD_{dermal}=\frac{C_{water}\times SA\times K_{p}\times ET\times EF\times ED\times 10^{-3}}{BW\times AT}$$

(3) $$ADD_{ingestion}=\frac{C_{water}\times IR\times EF\times ED}{BW\times AT}$$

where *ADD_ingestion_* and *ADD_dermal_* indicate the exposure dose of direct intake and skin absorption, respectively. These parameters were shown in Table S1 and *K_p_* is available in Table S2.

Furthermore, the hazard index (HI) represents the degree of harm to human health from heavy metal(loid)s. HI > 1 indicates a greater likelihood of harmful health risks, and HI < 1 indicates no appreciable harmful effects of heavy metal(loid)s on human health [39].

(4) HQ=ADD/RfD

(5) HI=∑HQs

where HQ is the hazard quotient, HI is the hazard index, and RfD represents the reference dose (Table S2).

(6) CR=∑ADD×SF

where CR indicates the carcinogenic risks and *SF* indicates the slope factor of heavy metal(loid)s (Table S2).

According to USEPA [41], the carcinogenic risks were divided into three levels according to CR value: CR < 10−6 (negligible); 10−6 < CR < 10−4 (acceptable); CR > 10−4 (high risk).

#### 2.3.3. Pollution Sources of Heavy Metal(loid)s

In the present research, source apportionment of pollutants uses the positive matrix factor (PMF) model [42]. PMF is a multivariate factor analysis tool that can distinguish different sources in a mixture and calculate their contributions to each species. The PMF model decomposes the original matrix X into a residual matrix (*E*), factor load matrix (*F*), and factor score matrix (*G*). According to the user guide of EPA PMF 5.0, the calculation formula is as follows:

(7) $$X_{ij}=\overset{p}{\underset{k=1}{\sum }}\left(G_{ik}\times F_{kj}\right)+E_{ij}$$

where *X* is the content matrix of the sample, *G* is the contribution of the factor to each sample, *F* is the species distribution for each source, *E* is the residual matrix, *i* indicates the *i*-th sample, *j* indicates the *j* -th element, and *k* indicates the *k*-th potential source.

The original matrix *X* is decomposed by the PMF model. The main objective of the model is to optimize and minimize the objective function *Q*. The calculation formula of the objective function *Q* is:

(8) $$Q=\sum_{i=1}^{n}\sum_{j=1}^{m}{\left(\frac{E_{ij}}{U_{ij}}\right)}^{2}$$

where *U_ij_* represents the uncertainty of the *j*-th chemical composition of the *i*-th sample. The uncertainty is defined as:

(9) $$U_{ij}=\left\{\begin{array}{c} \;\left(5/6\right)\times MDL,\;\left(c\leq MDL\right)\\ \sqrt{{\left(Error\;Fraction\times concentration\right)}^{2}+{\left(0.5\times MDL\right)}^{2}},\;\left(c\leq MDL\right) \end{array}\right.$$

where *c* represents the concentration of chemical species and *MDL* represents the detection limit of the specific method.

### 2.4. Statistical Analysis

In this study, the statistical analysis of eleven heavy metal(loid)s was performed using SPSS 23.0 software (Chicago, IL, USA), including minimum, maximum, mean, and standard deviation. EPA PMF 5.0 model was adopted to complete the source apportionment of heavy metal(loid)s. ArcGIS10.7 (RedLands, CA, USA) was applied in map delineation.

## 3. Results and Discussion

### 3.1. Physicochemical Analysis of Heavy Metal(loid)s Concentrations in Surface Water in the Study Area

The results of water physicochemical parameters are presented in Table 2. The pH values of the river water are between 6.85 and 8.73, with a mean value of 7.90, indicating that the pH of the Lijiang River Basin has little change and is mostly weakly alkaline. The temperature of the river water ranged from 14.76 to 21.35 °C, with a mean value of 17.71 °C. The temperature of the river water does not vary much in different seasons. The total dissolved solids (TDS) in the study areas fluctuated widely, with a range of 18.51 to 180.40 mg·L^−1^ and a mean value of 100.83 mg·L^−1^. Excessive TDS could cause water temperature to rise, hinders photosynthesis, and reduces water clarity [20]. Dissolved oxygen (DO) concentration varied between 6.03 and 14.01 mg·L^−1^, with a mean value of 9.74 mg·L^−1^, which is better than Class I water quality according to the National Environmental Quality Standards for Surface Water (GB3838-2002) in the Li River Basin. Electrical conductivity (EC) of river water ranged from 36.67 to 419.20 µs·cm^−1^, with EC values above the WHO guideline (200 µs·cm^−1^) at most sampling points, indicating high EC values in the region; a higher conductivity reflects higher water pollution [20], which is likely due to the surface runoff carrying minerals and organic matters into the river [43].

### 3.2. Time Distribution Characteristics of Heavy Metal(loid)s Concentrations in Surface Water in the Study Area

The variation of heavy metal(loid)s concentrations in surface water during the wet season and the dry season in the research region are shown in Table 3, along with the National Environmental Quality Standards for Surface Water (GB3838-2002) and WHO [38,44] for comparison. The mean concentrations of heavy metal(loid)s in surface water in the wet and dry seasons were ranked as: Al > Mn > Zn > Ni > Cd > Cr > Cu > As > Hg = Pb > Co, with the mean concentration of Hg being higher than the national Class II surface water environmental quality standard. The mean concentrations of Al, Pb, Zn, Cr, Co, Mn, and As in the wet season were higher than in the dry season, while those of Cu, Cd, Ni, and Hg were higher in the dry season. The variance may result from the interaction of surface runoff and wet and dry deposition. During the dry season, with the reduction of water flow rate, the dry (wet) deposition of heavy metal(loid)s accumulate on the surface. In the wet season, with the increase in water flow, heavy metal(loid)s entered the river through surface runoff [45].

During the wet season, the mean concentrations of heavy metal(loid)s Al, Cu, Pb, Zn, Cr, Ni, Co, Mn, and As were 69.73, 0.50, 0.39, 6.45, 1.10, 1.30, 0.26, 33.53, and 0.56 ug·L^−1^, which did not exceed the permitted limits by the national Class II surface water environmental quality standard or WHO. Although the mean value of Al did not exceed the standard, its maximum value (254 ug·L^−1^) exceeded the limit of the WHO drinking water quality standard, which may be related to the industrial area of the study area [29]. During the dry season, the mean concentrations of heavy metal(loid)s Al, Cu, Zn, Cr, Ni, Co, Cd, Mn, As, and Hg were 3.91, 1.42, 1.53, 1.00, 1.31, 0.09, 2.20, 3.70, 0.16, and 0.39 ug·L^−1^, respectively, with all heavy metal(loid)s concentrations, except for Hg, being within the national Class II surface water environmental quality standard; this is likely because there are highly intensive agricultural production areas upstream, and the use of chemical fertilizers and pesticides led to the accumulation of Hg in water bodies [46].

The coefficient of variation (CV) indicates the degree of dispersion of the elements between each sample, and the magnitude of the CV value was proportional to the degree of dispersion. According to the grading of the coefficient of variation in relevant studies [47], the coefficient of variation of Cd was 0.02, belonging to low variability (CV < 0.16), while those for Al, Cu, Pb, Zn, Cr, Ni, Co, Mn, As, and Hg belonged to high variability (CV > 0.36). Overall, the majority of the eleven heavy metal(loid)s were highly variable, indicating that surface water heavy metal(loid)s could be influenced by anthropogenic activities in the study area.

The concentrations of heavy metal(loid)s in surface water of the Lijiang River were compared with other rivers around the world and are given in Table 4. It is evident that the concentrations of Al, Cu, Pb, Zn, Ni, Co, As, and Hg were lower than in all other rivers. For other heavy metal(loid)s, Cu concentration was higher than in Bogacayi, Turkey. Cr concentrations were higher than in the Pardo River, Brazil. Cd concentrations were higher than in Bogacayi, Pardo River, Old Brahmaputra, and Lake Chaohu Basin. Mn concentration was higher than in Bogacayi, Turkey. The higher accumulation of these heavy metal(loid)s in surface water is attributed to the influence of anthropogenic sources (agricultural activities and industrial wastewater), directly or indirectly [29]. In addition, the analysis, with results shown in Table 3, found some accumulation of Al and Hg in different seasons, while in other rivers that were considered, the concentration of metals was much lower than observed. Therefore, our results were comparable to the uncontaminated river.

### 3.3. Spatial Distribution Characteristics of Heavy Metal(loid)s Concentrations in Surface Water in the Study Area

**Figure 3:**
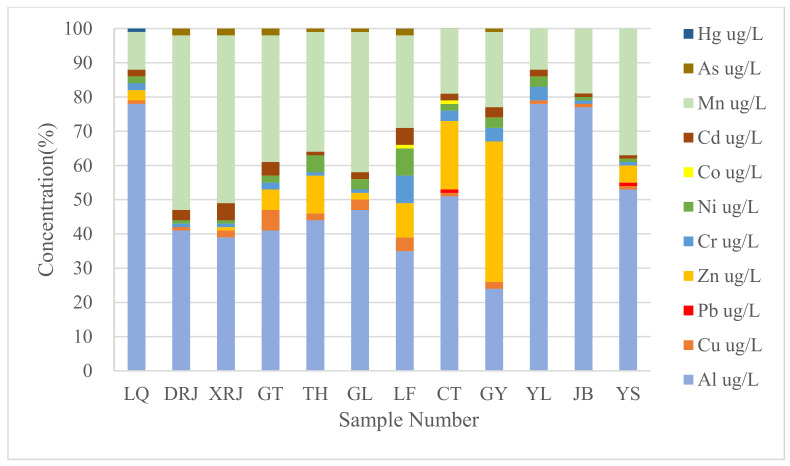
Spatial distribution of heavy metal(loid)s in surface water.

Observing the spatial distribution of heavy metal(loid)s in surface water is necessary for determining the level of contamination, as this allows areas with higher heavy metal(loid)s concentrations to be recognized and possible sources to be identified. The spatial distribution of heavy metal(loid)s was shown in Figure 3, whereby a significant spatial variation could be observed between the upstream (LQ, DRJ, and XRJ), midstream (GT, TH, GL, LF, and CT), and downstream (GY, YL, JB, and YS) areas. Some heavy metal(loid)s, especially Cu, Zn, Cr, Cd, Ni, Co, Cd, and As, exhibited a higher concentration in the midstream area compared with upstream and downstream areas. However, the midstream passes through the downtown area of the city [29], and the characteristics of spatial distribution could originate from environmental contamination caused by anthropogenic activities, such as industrial activities, transportation, and agricultural activities. Conversely, Mn and Hg showed heterogeneity in the upstream area, which might be correlated with highly intensive agricultural production. Al and Pb showed a slight variation in the downstream area, possibly influenced by industrial activities. Overall, most heavy metal(loid)s were distributed mainly in the midstream area, which was consistent with the results of Xiao et al. [29]. These findings are associated with the input of heavy metal(loid)s contamination from anthropogenic sources, likely to be mainly industrial activities, traffic sources, pesticides, and fertilizers [51].

### 3.4. Surface Water Quality

**Figure 4:**
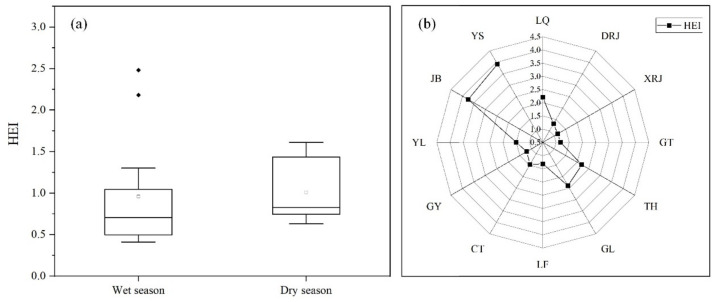
Seasonal (**a**) and spatial (**b**) variation of HEI.

Figure 4 shows the seasonal and spatial variation of HEI. On the seasonal scale (Figure 4a,b), the HEI was significantly higher in the dry season (mean of 1.01) than in the wet season (mean of 0.96), which was due to the larger rainfall in the wet season and the dilution effect resulting in lower values of HEI. In view of the HEI classification, the water quality of the wet season was “poor”, and the dry season was “slightly affected”. The status “poor” was the major status in the wet season, which accounted for 75.00% of all sampling points. There were 16.67% and 8.33% sites with “slightly affected” and “moderately affected” status, respectively. The status “poor” was the major status in the dry season, which accounted for 66.67% of all sampling sites, and only 33.33% of the site was in “slightly affected” status.

Additionally, the HEI makes a significant difference in terms of space (Figure 3b), with a mean value of 1.96, and the contamination status was categorized as “slightly affected”. The mean values of HEI were 1.56 in the upstream area, 1.71 in the midstream area, and 2.59 in the downstream area, while the statuses were “slightly affected”, “slightly affected”, and “moderately affected”, respectively. The spatial range of the HEI was 1.15 to 3.92, and the maximum and minimum values were observed in downstream and upstream areas, respectively. In the upstream area, the primary water quality status was “slightly affected”, accounting for 33.33% of all sampling points, followed by “slightly affected” (66.67%). Among the sampling points in the midstream, 60.00% and 40.00% of sites were classified as “slightly affected” and “moderately affected”, respectively. In the downstream area, 50.00% of points were classified as “slightly affected”, and 50.00% were “moderately affected”. This may be due to the fact that downstream was a scenic area, which is affected the activities of boats and has an impact on the aquatic environment.

### 3.5. Pollution Sources of Heavy Metal(loid)s in Surface Water in the Study Area

In order to further analyze the sources of heavy metal(loid)s in the surface water of the study area, the PMF model was used to analyze their sources. During the operation of the model, 4~7 factors were selected to perform 20 iterations, respectively, which were calculated repeatedly according to species proportion residual, signal-to-noise ratio, correlation between observed value and predicted value, and Q value until the optimal number of factors was determined [47]. When the number of factors was 5, the difference of Q values was minimal and most of the residuals were between −3 and ~ 3. Therefore, the optimal number of factors for PMF source resolution in this study was 5.

**Figure 5:**
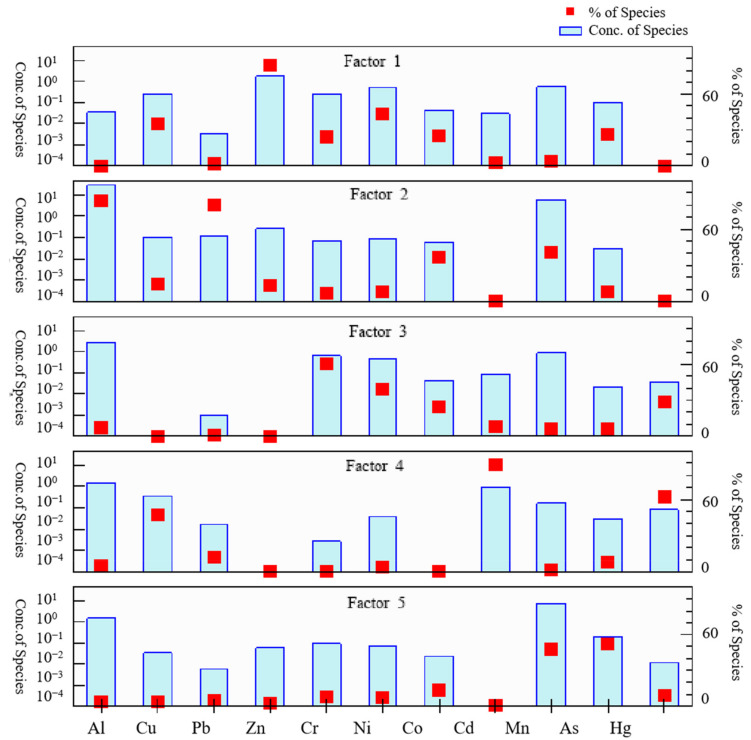
Contribution of heavy metal(loid)s PMF source analysis.

By analyzing the source of eleven heavy metal(loid)s, the following five main sources can be identified (Figure 5). The contribution rates of Factor 1 to Zn and Ni concentrations were 84.5% and 44.1%, respectively. Studies have shown that Zn and Ni are relevant to vehicles and were abundant in vehicle exhaust [47]. Zn can be used as a landmark element of traffic contamination sources since it may originate from traffic pollutants, such as motor vehicle brake pads, lubricant combustion, exhaust emissions, and tire wear [52]. The higher concentrations of Zn and Ni were distributed mainly in the midstream area, which was the area most influenced by urban activities [29]. It could be related to the dense population and heavy vehicle traffic around the sampling sites as well as to the various wastewaters, exhaust, and hull corrosion from tourist vessels, which might increase the concentrations of these heavy metal(loid)s in the midstream area [29,52]. Therefore, traffic could be considered as the source of Factor 1.

Factor 2 contributed to the concentrations of Al, Pb, Mn, and Co, with proportions of 84.0%, 80.7%, 41.4%, and 36.5%, respectively. The presence of industrial areas in the study area, including materials, chemical industry, electronic, and Guilin National Hightech Zone Yingcai Science and Technology Park, may likely be the anthropogenic source of these heavy metal(loid)s [29]. The contamination of Pb acid batteries, fuel combustion, and industrial emissions could also lead to the accumulation of Al, Pb, Mn, and Co in water bodies [20,53]. Hence, the above results showed that industrial activities could be termed as the source of Factor 2.

Factor 3 contributed 60.2% and 38.7% to the concentrations of Cr and Ni, respectively. The investigation revealed that the domestic waste was commonly deposited randomly in the rural areas of the Lijiang River basin, and the adjacent waters were also foul-smelling and black [52]. The study found that the accumulation of various garbage, household materials, cans, etc., could lead to the accumulation of Cr [53]. The wastes produced in human life all contain Cr and Ni, which were leached by rainwater, and Cr and Ni will enter the river along with the landfill leachate [54]. Therefore, we believe that Factor 3 was likely to be caused by domestic waste emissions.

Factor 4 was dominated by Cd (88.7%), Hg (62.4%), and Cu (47.1%). The above study found that higher concentrations of Hg were distributed mainly in the upstream area, where there were highly intensive agricultural production areas. Research shows that Hg is the primary ingredient in pesticides and fertilizers, with volatility and migration [46]. Cd and Cu were generally used as marker elements for agricultural activities, such as the use of pesticides and fertilizers [55]. The water in the Li River basin irrigates the surrounding fertile land, so the pesticides and fertilizers applied to farmland could find their way back to the river, resulting in the accumulation of heavy metal(loid)s in water bodies [29]. This could be the main source of Factor 4.

Factor 5 contributed 52.4% and 47.9% to the concentrations of As and Mn, respectively. Related literature indicates that they are derived mainly from the natural geological background such as rock weathering, soil erosion, etc. Under the action of rainfall, Co, Cr, As, and Ni may be transferred to bodies of water [3,56]. It has been found that As and Mn originated from natural geology, and their distributions in water bodies were likely correlated with the petrogenesis and higher weathering rate of carbonate rocks and minerals [28]. Thus, Factor 5 comes mainly from natural source dominated by rock weathering.

**Figure 6:**
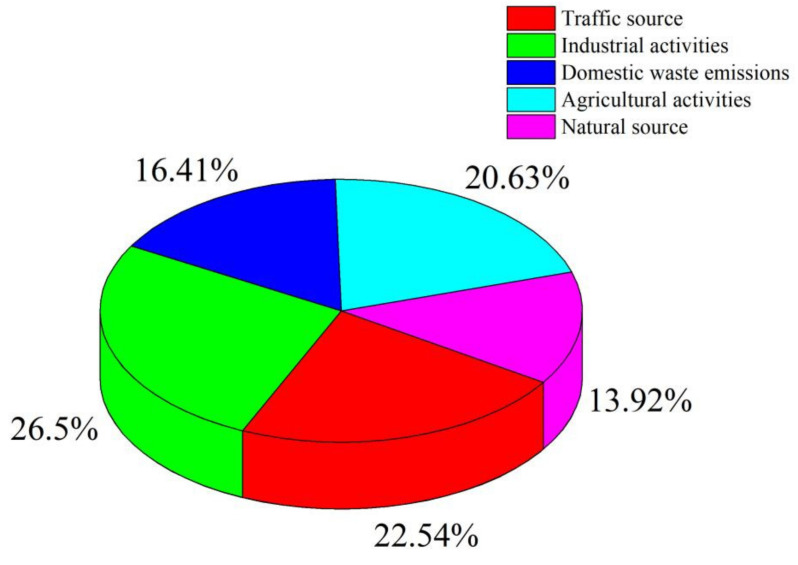
Contribution rate of different sources of heavy metal(loid)s.

The above study concluded that the main pollution sources of heavy metal(loid)s in surface water in the study area are industrial activities, traffic sources, agricultural activities, domestic waste emissions, and natural sources, with the combined contribution of these five sources being 26.50%, 22.54%, 20.63%, 16.41%, and 13.92% respectively (Figure 6). In conclusion, anthropogenic activities play a dominant role, with approximately 86.08% of the contamination being caused by anthropogenic factors, with industrial activities, traffic sources, and agricultural activities being the main sources of heavy metal(loid)s contamination in the study area.

### 3.6. Human Health Risk Assessment

#### 3.6.1. Human Health Risk Assessment of Heavy Metal(loid)s

**Figure 7:**
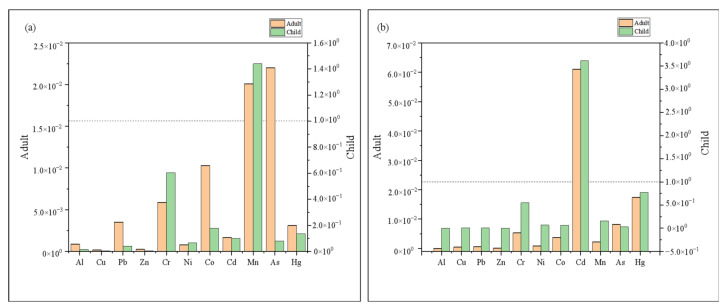
HI for adults and children during the wet (**a**) and dry (**b**) seasons.

**Figure 8:**
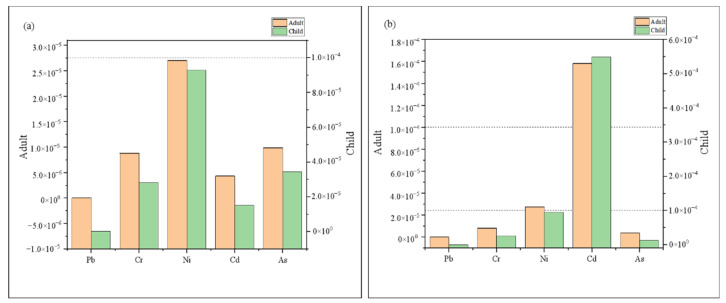
CR for adults and children during the wet (**a**) and dry (**b**) seasons.

Figure 7 and Figure 8 and Table S3 presented the mean values of HQ, HI, and CR for the oral and dermal pathways for wet and dry seasons relating to adults and children, respectively. In the wet season, HI and HQingestion of all elements except Mn for children were smaller than 1 (Table S3, Figure 7), suggesting that Mn posed serious health concerns to children via oral intake, while other elements via oral intake had no or little health threat. In addition, HQdermal of 11 elements (Al, Cu, Pb, Zn, Cr, Ni, Co, Cd, Mn, As, and Hg) for adults and children were all below unity, indicating that these metals posed little hazards via dermal absorption. Among these trace elements, Pb, Cr, Ni, Cd, and As are carcinogenic (Table S4, Figure 8). The CR values of Pb were all < 10^−6^, pointing to extremely low cancer risk, which is generally unlikely to cause cancer [2]. The CR values between 10^−6^ and 10^−4^ for Cr, Ni, Cd, and As, indicating acceptable risk. Furthermore, it should be noted that HI and CR values were higher in children than with adults, which was more in line with the study by Wu et al. [2]. Growing and developing children have a higher exposure sensitivity per unit of weight due to strong gastrointestinal absorption, higher respiratory rate, and some behavioral habits [2,57].

In the dry season, the HI and HQingestion values of Cd for children were >1, which posed a greater chance of harmful human health risks to children. HQdermal was consistent with the wet season, indicating no or little health threat through dermal absorption. The CR values of Pb were all <10^−6^, pointing to extremely low cancer risk. The CR values between 10^−6^ and 10^−4^ for Cr, Ni, and As, indicating acceptable risk. The CRingestion values of Cd for adults and children were >10^−4^, demonstrating high risk, which cannot be ignored. Consequently, populations of the study area are vulnerable to increased cancer risk due to Cd exposure in surface water, mainly through the direct intake route. Cd exposure could influence the growth rate of minority populations in the study area [57]. The children’s populations in the study area might also be susceptible to osteoporosis and anemia. In addition, the Intelligence Quotient levels of the study children may be reduced due to Cd exposure [57,58].

**Figure 9:**
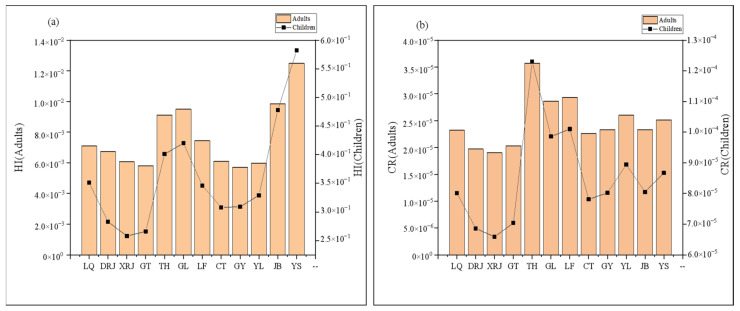
Spatial distribution of heavy metal(loid)s HI (**a**) and CR (**b**) in surface water.

Seasonally, HI and CR values in the dry season were significantly higher than those in the wet season, regarding both adults and children. Spatially (Figure 9), the highest values of adult and child HI appear at the YS point in the downstream area, which is a tourist attraction and may be influenced by tourism transportation. However, the CR values were significantly higher in the midstream area, regardless of adults and children, which was consistent with the above study on the spatial distribution of heavy metal(loid)s concentrations, which may be influenced by industrial activities, transportation, fertilizers, and pesticides.

However, the US EPA highlighted that there was uncertainty of risk characterization [59]. Additionally, the exposure parameters applied in the research were from the US EPA, which might not be specific to China, and the exposure parameters varied considerably between populations in different regions [27]. Since this study basically collected 12 points in the upper, middle, and lower reaches of the Lijiang River, the actual drinking water for residents was treated by the waterworks, and the concentration of heavy metal(loid)s may be lower than that of the Lijiang River water samples, which actually overestimated the risk of heavy metal(loid)s exposure. Therefore, the research on the health risk assessment of heavy metal(loid)s in drinking water sources in the Lijiang River Basin needs to be further explored.

#### 3.6.2. Source-Oriented Health Risk Assessment of Heavy Metal(loid)s

**Figure 10:**
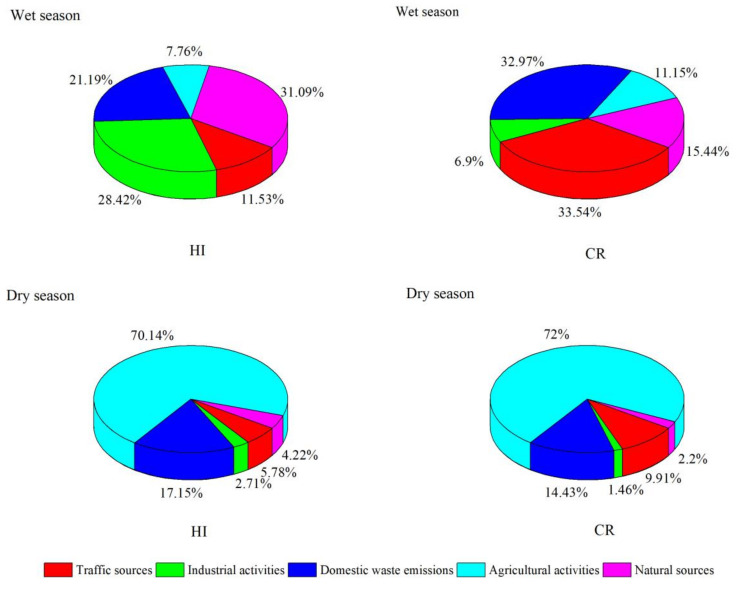
Cumulative probability of HI and CR from the identified pollution sources.

The non-carcinogenic and carcinogenic health risks of different sources of heavy metal(loid)s in the study area can be obtained by combining the contaminant source contributions of heavy metal(loid)s with the human health risk assessment model (Figure 10). During the wet season, the pollution source that contributes to the most to HI is natural source (31.09%), followed by industrial activities (28.42%), domestic waste emissions (21.19%), traffic source (11.53%), and agricultural sources (7.76%). The largest contribution to CR came from traffic sources (33.54%), followed by domestic waste emissions (32.97%), natural sources (15.44%), agricultural sources (11.15%), and industrial activities (6.90%). The variations in the source contribution were attributed to the different toxicity factors of heavy metal(loid)s [40]. The contribution of traffic sources was the largest, indicating that human activities have caused health risks to a large extent.

During the dry season, the order of the HI was agricultural sources > domestic waste emissions > traffic sources > natural sources > industrial activities. The order of the CR was agricultural sources > domestic waste emissions > traffic sources > natural sources > industrial activities. Agricultural sources contribute the most to HI and CR, which was attributed mainly to the pervasive use of pesticides and fertilizers [60]. Zeng et al. also found that the heavy use of herbicide pesticides leads to higher environmental and health risks [61]. Hence, it is also necessary to control the application of fertilizers and pesticides to protect human health.

In summary, anthropogenic activities have a significant effect on heavy metal(loid)s in the study area. Moreover, agricultural activities and transportation should be appropriately adjusted to protect the stability of the ecosystem and the health of the residents in the Lijiang River Basin.

## 4. Conclusions

In conclusion, the water quality status, sources, and health risks of heavy metal(loid)s were identified in this study using the Lijiang River as an example. The water quality at the 12 sampling points in the study area was satisfactory in terms of pH, total dissolved solids, and dissolved oxygen, and only the electrical conductivity value was higher. Concentrations of heavy metal(loid)s demonstrated great seasonality, the mean concentrations of Al, Pb, Zn, Cr, Co, Mn, and As were higher in the wet season, while Cu, Cd, Ni, and Hg elements were higher in the dry season. There were also differences in space, with most heavy metal(loid)s distributed mainly in the midstream area. The HEI index indicated that the main water quality status was “slightly affected” in the study area. The PMF model was employed to identify and quantify the potential pollution sources of heavy metal(loid)s, and five potential pollution sources were obtained: industrial activities, traffic sources, agricultural activities, domestic waste emissions, and natural resources, with contribution rates of 26.50%, 22.54%, 20.63%, 16.41%, and 13.92%, respectively. Concerning risk assessment, Mn and Cd had the highest HI values for children, which accounted for 1.44 × 10^0^ and 3.62 × 10^0^, respectively. Cd had the highest contribution to CR for adults and children, which was 1.58 × 10^−4^ and 5.49 × 10^−4^, respectively. The source-oriented risk assessment indicated that the natural sources had the highest contribution to the HI, with 31.09%, and transport sources contributed the most to CR, with 33.54% during the wet season. In the dry seasons, the contribution rate of agricultural sources to HI and CR was the largest, accounting for 70.14% and 72%, respectively. This study used a probabilistic risk estimation method in assessing health risks, providing evidence for identifying the emphasis on risk heavy metal(loid)s in further risk control, which could be used to assess local contamination levels and formulate reasonable ecological protection measures.

The source-oriented health risk assessment used in this study is a bridge between potential pollution sources and health risk assessment, quantifying the health risks of residents from each type of pollution source in the study area. However, the concentrations of heavy metals (loid)s in the water samples of this study may be higher than the actual drinking water of the residents. Therefore, there is a need for further research on source- oriented health risk assessment of heavy metals (loid) in drinking water sources in the Lijiang River basin. 

## Figures and Tables

**Table 1:** Distribution of sampling points.

Areas	Sampling Points	Abbreviations
Upstream area	Lingqu	LQ
	Darong River	DRJ
	Xiaorong River	XRJ
Midstream area	Gantang River	GT
	Taohua River	TH
	Guilin Section	GL
	Liangfeng River	LF
	Chaotian River	CT
Downstream area	Guangyan	GY
	Yangshuo Section	YS
	Yulong River	YL
	Jinbao River	JB

**Table 2:** Physicochemical parameters of surface water in the Lijiang River Basin.

Season		pH	T/°C	TDS/mg·L^−1^	DO/mg·L^−1^	EC/µs·cm^−1^
Wet season	Max	8.22	21.35	180.40	10.30	357.30
	Min	6.85	14.88	18.51	6.03	36.67
	Mean	7.66	19.43	92.42	8.95	183.62
Dry season	Max	8.73	17.15	144.70	14.01	419.20
	Min	7.42	14.76	75.40	7.27	41.40
	Mean	8.13	16.01	109.23	10.53	233.93

**Table 3:** Statistical results of surface water heavy metal(loid)s concentrations in the study area.

Elements		Al	Cu	Pb	Zn	Cr	Ni	Co	Cd	Mn	As	Hg
Wet season	Mean	69.73	0.50	0.39	6.45	1.10	1.30	0.26	ND	33.53	0.56	ND
	CV	1.03	0.68	1.58	1.41	0.46	0.75	0.66	-	0.96	0.47	-
Dry season	Mean	3.91	1.42	ND	1.53	1.00	1.31	0.09	2.20	3.70	0.16	0.39
	CV	1.05	0.78	-	1.59	0.60	0.78	0.74	0.05	2.50	1.39	0.83
National Class II surface water environmental quality standard		-	1000	10	1000	50	-	-	5	-	50	0.05
Standard (WHO)		200	2000	10	-	50	20	-	3	400	10	6

“-” denotes no relevant data; “ND” Not detected.

**Table 4:** Variation in heavy metal(loid)s concentration of different rivers across the world.

River	Bogacayi, Turkey	Sirsa River, India	Haraz River, Iran	Pardo River, Brazil	Old Brahmaputra River, Bangladesh	Lake Chaohu Basin, China	Lijiang River, China
Al	-	-	-	-	6870	-	36.82
Cu	0.92	27.0	13.25	5.05	120	1.63	0.96
Pb	0.48	17.9	4.40	2.54	110	0.41	0.20
Zn	-	96.3	52.75	12.45	10	233.39	3.99
Cr	3.20	35.5	-	0.52	10	1.15	1.05
Ni	3.47	64.8	-	5.70	440	2.35	1.30
Co	ND	-	-	-	200	-	0.17
Cd	0.23	2.6	2.65	0.05	1	0.02	1.10
Mn	0.62	313.9	116	36.4	1440	38.85	18.61
As	0.43	5.5	55.35	2.14	-	1.50	0.36
Hg	ND	-	-	ND	1	-	0.20
Reference	[13]	[14]	[48]	[49]	[50]	[39]	Present study

“-” denotes no relevant data.

## Data Availability

Not applicable.

## Supplementary Materials

The following supporting information can be downloaded at: https://www.mdpi.com/article/10.3390/toxics10120726/s1, Table S1: Distributions of parameters for the health risk assessment of heavy metal(loid)s.; Table S2: Reference dose (RfD) and dermal permeability coefficient in samples (Kp), and slope factor (SF) of heavy metal(loid)s used for health risk assessment. Table S3: Summary statistics for non-carcinogenic risks of children and adults. Table S4: Summary statistics for carcinogenic risks of children and adults.
