# Supplementary material for: Health Risk Assessment Based on Source Identification of Heavy Metal(loid)s: A Case Study of Surface Water in the Lijiang River, China
Source: Toxics. 2022 Nov 25;10(12):726. doi: 10.3390/toxics10120726 (PMC9783363; doi:10.3390/toxics10120726)
Supplement: Supplementary file 1 [file toxics-10-00726-s001.zip › toxics-2027879-supplementary.pdf]

toxics MDPI

Supplementary Materials: Health Risk Assessment Based on Source Identification of Heavy Metal(loid)s: A Case Study of Surface Water in the Lijiang River, China

Yu Wang, Cunlin Xin, Shi Yu, Yincai Xie, Wanjun Zhang and Rongjie Fu

Table S1. Distributions of parameters for the health risk assessment of heavy metal(loid)s.

Symbol	Parameter	Units	Distribution		References
			Adult	Child	
C_{water}	average concentration	μg/L			
IR	intake rate	L/day	2	0.64	[1]
EF	exposure frequency	days/year	350	350	[1]
ED	duration of exposure	years	30	6	[1]
SA	exposed area of skin	cm^2	1,8000	6600	[1]
ET	exposure time	h/day	1	0.58	[1]
BW	body weight	kg	70	15	[1]
AT	average time	days	2,5550	2190	[1]

Table S2. Reference dose (RfD) and dermal permeability coefficient in samples (Kp), and slope factor (SF) of heavy metal(loid)s used for health risk assessment.

Elements	$RfD_{ingestion}$(μg/kg/day)	RfD_{dermal}(μg/kg/day)	Kp (cm/h)	SF_{ing} $(mg/kg/d)^{-1}$	SF_{der} $(mg/kg/d)^{-1}$
Mn	24	0.96	1×10^{-3}		
Ni	20	0.8	2×10^{-4}	1.7	42.5
Pb	1.4	0.42	1×10^{-4}	8.5×10^{-3}	0.073
Zn	300	60	6×10^{-4}		
As	0.3	0.285	1×10^{-3}	1.5	1.5
Cr	3	0.075	1×10^{-3}	0.5	20
Cd	0.5	0.025	1×10^{-3}	6.1	0.38
Cu	40	8	1×10^{-3}		
Co	0.3	0.06	4×10^{-4}		
Hg	0.3	0.021	1×10^{-3}		
Al	1000	200	1×10^{-3}		

Table S3. Summary statistics for non-carcinogenic risks of children and adults.

Elements		$HQ_{ingestion}$		HQ_{dermal}		HI	
		Adult	Child	Adult	Child	Adult	Child
Wet season	Al	8.19×10^{-4}	1.43×10^{-2}	3.68×10^{-5}	8.53×10^{-5}	8.56×10^{-4}	1.44×10^{-2}
	Cu	1.47×10^{-4}	2.56×10^{-3}	6.62×10^{-6}	1.53×10^{-5}	1.54×10^{-4}	2.58×10^{-3}
	Pb	3.48×10^{-3}	4.04×10^{-2}	1.04×10^{-5}	2.42×10^{-5}	3.49×10^{-3}	4.05×10^{-2}
	Zn	2.65×10^{-4}	4.62×10^{-3}	7.16×10^{-6}	1.66×10^{-5}	2.72×10^{-4}	4.64×10^{-3}
	Cr	4.32×10^{-3}	6.02×10^{-1}	1.55×10^{-3}	3.60×10^{-3}	5.87×10^{-3}	6.05×10^{-1}
	Ni	7.61×10^{-4}	6.63×10^{-2}	3.42×10^{-5}	7.93×10^{-5}	7.95×10^{-4}	6.64×10^{-2}
	Co	1.01×10^{-2}	1.76×10^{-1}	1.82×10^{-4}	4.21×10^{-4}	1.03×10^{-2}	1.77×10^{-1}
	Cd	1.41×10^{-3}	9.82×10^{-2}	2.54×10^{-4}	5.87×10^{-4}	1.66×10^{-3}	9.88×10^{-2}
	Mn	1.64×10^{-2}	1.43×10^{0}	3.69×10^{-3}	8.55×10^{-3}	2.01×10^{-2}	1.44×10^{0}
	As	2.18×10^{-2}	7.98×10^{-2}	2.06×10^{-4}	4.77×10^{-4}	2.20×10^{-2}	8.03×10^{-2}

	Hg	2.74×10^{-3}	1.36×10^{-1}	3.52×10^{-4}	8.16×10^{-4}	3.09×10^{-3}	1.37×10^{-1}
Dry season	Al	4.60×10^{-5}	8.02×10^{-4}	2.07×10^{-6}	4.80×10^{-6}	4.81×10^{-5}	8.07×10^{-4}
	Cu	4.18×10^{-4}	7.28×10^{-3}	1.88×10^{-5}	4.35×10^{-5}	4.37×10^{-4}	7.32×10^{-3}
	Pb	5.87×10^{-4}	6.82×10^{-3}	1.76×10^{-6}	4.08×10^{-6}	5.89×10^{-4}	6.82×10^{-3}
	Zn	7.54×10^{-5}	1.31×10^{-3}	2.04×10^{-6}	4.71×10^{-6}	7.74×10^{-5}	1.32×10^{-3}
	Cr	3.91×10^{-3}	5.45×10^{-1}	1.41×10^{-3}	3.26×10^{-3}	5.32×10^{-3}	5.48×10^{-1}
	Ni	7.71×10^{-4}	6.72×10^{-2}	3.47×10^{-5}	8.04×10^{-5}	8.06×10^{-4}	6.73×10^{-2}
	Co	3.70×10^{-3}	6.44×10^{-2}	6.66×10^{-5}	1.54×10^{-4}	3.76×10^{-3}	6.46×10^{-2}
	Cd	5.17×10^{-2}	3.60×10^{0}	9.30×10^{-3}	2.15×10^{-2}	6.10×10^{-2}	3.62×10^{0}
	Mn	1.81×10^{-3}	1.58×10^{-1}	4.08×10^{-4}	9.44×10^{-4}	2.22×10^{-3}	1.59×10^{-1}
	As	8.11×10^{-3}	2.97×10^{-2}	7.68×10^{-5}	1.78×10^{-4}	8.18×10^{-3}	2.99×10^{-2}
	Hg	1.54×10^{-2}	7.68×10^{-1}	1.98×10^{-3}	4.59×10^{-3}	1.74×10^{-2}	7.73×10^{-1}

Table S4. Summary statistics for carcinogenic risks of children and adults.

**Elements**		**$CR_{ingestion}$**		**CR_{dermal}**		**CR**	
		**Adult**	**Child**	**Adult**	**Child**	**Adult**	**Child**
Wet season	Pb	4.14×10^{-8}	1.44×10^{-7}	3.20×10^{-10}	7.42×10^{-10}	4.18×10^{-8}	1.45×10^{-7}
	Cr	6.48×10^{-6}	2.26×10^{-5}	2.33×10^{-6}	5.40×10^{-6}	8.81×10^{-6}	2.80×10^{-5}
	Ni	2.59×10^{-5}	9.01×10^{-5}	1.16×10^{-6}	2.70×10^{-6}	2.70×10^{-5}	9.28×10^{-5}
	Cd	4.30×10^{-6}	1.50×10^{-5}	2.41×10^{-6}	5.58×10^{-9}	4.30×10^{-6}	1.50×10^{-5}
	As	9.79×10^{-6}	3.41×10^{-5}	8.81×10^{-8}	2.04×10^{-7}	9.88×10^{-6}	3.43×10^{-5}
Dry season	Pb	6.99×10^{-9}	2.43×10^{-8}	5.40×10^{-11}	1.25×10^{-10}	7.04×10^{-9}	2.45×10^{-8}
	Cr	5.87×10^{-6}	2.04×10^{-5}	2.11×10^{-6}	4.89×10^{-6}	7.98×10^{-6}	2.53×10^{-5}
	Ni	2.62×10^{-5}	9.14×10^{-5}	1.18×10^{-6}	2.73×10^{-6}	2.74×10^{-5}	9.41×10^{-5}
	Cd	1.58×10^{-4}	5.49×10^{-4}	8.84×10^{-8}	2.05×10^{-7}	1.58×10^{-4}	5.49×10^{-4}
	As	3.65×10^{-6}	1.27×10^{-5}	3.28×10^{-8}	7.60×10^{-8}	3.68×10^{-6}	1.28×10^{-5}

References

1. Wu, H.H.; Xu, C.B.; Wang, J.H.; Xiang, Y.; Ren, M.; Qie, H.T.; Zhang, Y.J.; Yao, R.H.; Li, L.; Lin, A.J. Health risk assessment based on source identification of heavy metals: A case study of Beiyun River, China. *Ecotox. Environ. Safe.* **2021**, *213*, 112046. https://doi.org/10.1016/j.ecoenv.2021.112046.
